# HVGH: Unsupervised Segmentation for High-Dimensional Time Series Using Deep Neural Compression and Statistical Generative Model

**DOI:** 10.3389/frobt.2019.00115

**Published:** 2019-11-20

**Authors:** Masatoshi Nagano, Tomoaki Nakamura, Takayuki Nagai, Daichi Mochihashi, Ichiro Kobayashi, Wataru Takano

**Affiliations:** ^1^Department of Mechanical Engineering and Intelligent Systems, The University of Electro-Communications, Tokyo, Japan; ^2^Graduate School of Engineering Science, Osaka University, Osaka, Japan; ^3^Artificial Intelligence Exploration Research Center, The University of Electro-Communications, Tokyo, Japan; ^4^Department of Statistical Inference and Mathematics, The Institute of Statistical Mathematics, Tokyo, Japan; ^5^Advanced Sciences, Graduate School of Humanities and Sciences, Ochanomizu University, Tokyo, Japan; ^6^Center for Mathematical Modeling and Data Science, Osaka University, Osaka, Japan

**Keywords:** motion segmentation, Gaussian process, variational autoencoder, hidden semi-Markov model, motion capture data, high-dimensional time-series data

## Abstract

Humans perceive continuous high-dimensional information by dividing it into meaningful segments, such as words and units of motion. We believe that such unsupervised segmentation is also important for robots to learn topics such as language and motion. To this end, we previously proposed a hierarchical Dirichlet process–Gaussian process–hidden semi-Markov model (HDP-GP-HSMM). However, an important drawback of this model is that it cannot divide high-dimensional time-series data. Furthermore, low-dimensional features must be extracted in advance. Segmentation largely depends on the design of features, and it is difficult to design effective features, especially in the case of high-dimensional data. To overcome this problem, this study proposes a hierarchical Dirichlet process–variational autoencoder–Gaussian process–hidden semi-Markov model (HVGH). The parameters of the proposed HVGH are estimated through a mutual learning loop of the variational autoencoder and our previously proposed HDP-GP-HSMM. Hence, HVGH can extract features from high-dimensional time-series data while simultaneously dividing it into segments in an unsupervised manner. In an experiment, we used various motion-capture data to demonstrate that our proposed model estimates the correct number of classes and more accurate segments than baseline methods. Moreover, we show that the proposed method can learn latent space suitable for segmentation.

## 1. Introduction

Humans perceive continuous high-dimensional information by dividing it into meaningful segments, such as words and units of motion. For example, we recognize words by segmenting speech waves, and we perceive particular motions by segmenting continuous motion. Humans learn words and motions by appropriately segmenting continuous information without explicit segmentation points. We believe that such unsupervised segmentation is also important for robots, in order for them to learn language and motion.

In this paper, we define the segments as arbitrary temporal patterns that appear multiple times in the time-series data. Additionally, we have proposed a method to extract the segments by capturing such a nature stochastically. One of our previous methods is the hierarchical Dirichlet process–Gaussian process–hidden semi-Markov model (HDP-GP-HSMM) (Nagano et al., 2018). HDP-GP-HSMM is a non-parametric Bayesian model that is a hidden semi-Markov model, the emission distributions of which are Gaussian processes (MacKay, 1998), and it facilitates the segmentation of time-series data in an unsupervised manner. In this model, segments are continuously represented using a Gaussian process. Moreover, the number of segmented classes can be estimated using hierarchical Dirichlet processes (Teh et al., 2006). The Dirichlet processes assume an infinite number of classes. However, only a finite number of classes are actually used. This is accomplished by stochastically truncating the number of classes using a slice sampler (Van Gael et al., 2008).

**Figure 1:**
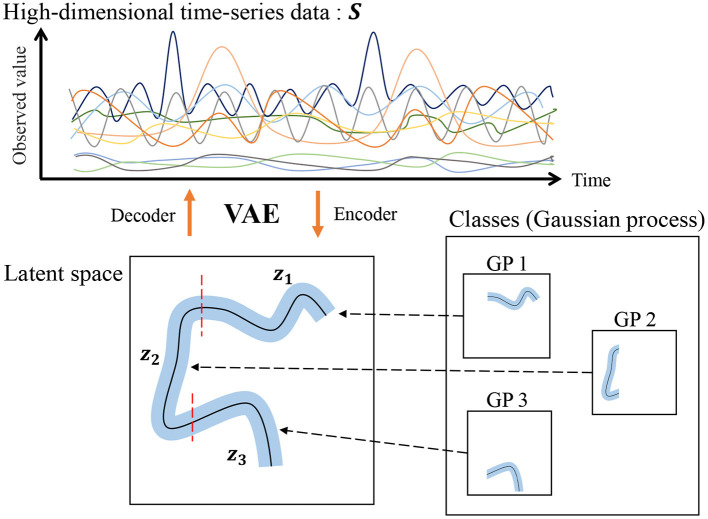
Overview of the generative process of the HVGH.

However, our HDP-GP-HSMM cannot handle high-dimensional data, and feature extraction is required to reduce the dimensionality in advance. Indeed, segmentation largely depends on this feature extraction, and it is difficult to extract effective features, especially in the case of high-dimensional data. To overcome this problem, this study introduces a variational autoencoder (VAE) (Kingma et al., 2013) to HDP-GP-HSMM. Thus, the model we propose in this paper is a hierarchical Dirichlet process–variational autoencoder–Gaussian process–hidden semi-Markov model (HVGH^1^). Figure 1 shows an overview of HVGH. The observation sequence is compressed and converted into a latent variable sequence by the VAE, and the latent variable sequence is divided into segments by HDP-GP-HSMM. Furthermore, parameters learned by HDP-GP-HSMM are used as the hyperparameters for the VAE. In this way, the parameters are optimized in a mutual learning loop, and appropriate latent space for segmentation can be learned by the VAE. In experiments, we evaluated the efficiency of the proposed HVGH using real motion-capture datasets. The experimental results show that HVGH achieves a higher accuracy compared to baseline methods.

Many studies on unsupervised motion segmentation have been conducted. However, heuristic assumptions are used in many of them (Lioutikov et al., 2015; Wächter et al., 2015; Takano et al., 2016). Wächter et al. proposed a method for segmenting object-manipulation motion in robots and used contact between the end-effector and the object as a segmentation clue (Wächter et al., 2015). Lioutikov et al. proposed a method that requires candidates for the segmentation points in advance (Lioutikov et al., 2015). In addition, the method proposed by Takano et al. used errors between the predicted and actual values as criteria for segmentation (Takano et al., 2016). Moreover, some methods use motion features such as the zero velocity of joint angles (Fod et al., 2002; Shiratori et al., 2004; Lin et al., 2012). However, this assumption typically induces over-segmentation (Lioutikov et al., 2015).

Furthermore, studies have proposed methods of detecting change points in time-series data in an unsupervised manner (Yamanishi et al., 2002; Lund et al., 2007; Liu et al., 2013; Haber et al., 2014). These are the methods of finding points with different fluctuations based on previous observations. Therefore, these methods assume that similar temporal patterns are repeated between the change points. On the other hand, in this study, we consider that the segments comprise not only repeated patterns but also an arbitrary pattern. Thus, the change points do not necessarily indicate the segment boundaries.

**Figure 2:**
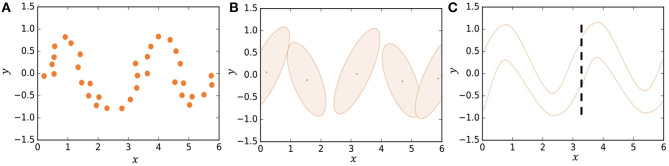
Representing a trajectory: **(A)** Observed data points, **(B)** representation by HMM, and **(C)** representation by HDP-GP-HSMM.

In some studies, segmentation is formulated stochastically using hidden Markov models (HMMs) (Beal et al., 2002; Fox et al., 2011; Taniguchi et al., 2011; Matsubara et al., 2014). However, it is difficult for HMMs to represent complicated motion patterns. Instead, we use Gaussian processes in our model, which is a type of non-parametric model that can better represent complicated time-series data compared to HMMs. Figure 2B shows how an HMM represents the trajectory of data points shown in Figure 2A. The HMM represents the trajectory using five Gaussian distributions. However, one can see that the details of the trajectory are lost. On the other hand, in HDP-GP-HSMM (Figure 2C), the trajectory can be represented continuously using two Gaussian processes (GPs). We confirmed that our GP-based model can estimate segments more accurately than HMM-based methods (Nagano et al., 2018).

In some studies, the number of classes is estimated by introducing a hierarchical Dirichlet process (HDP) into an HMM (Beal et al., 2002; Fox et al., 2007). An HDP is a method of estimating the number of classes by assuming an infinite number of classes. Fox et al. extended an HDP–HMM to develop a so-called sticky HDP-HMM, which prevents over-segmentation by increasing the self-transition probability (Fox et al., 2007).

Among methods of combining statistical models with neural networks, a method of classifying complicated data using a GMM and VAE was proposed (Johnson et al., 2016). In contrast, our proposed HVGH is a model that combines a statistical model with a VAE for segmenting high-dimensional time-series data.

## 2. Hierarchical Dirichlet Process–Variational Autoencoder–Gaussian Process–Hidden Semi-Markov Model (HVGH)

**Figure 3:**
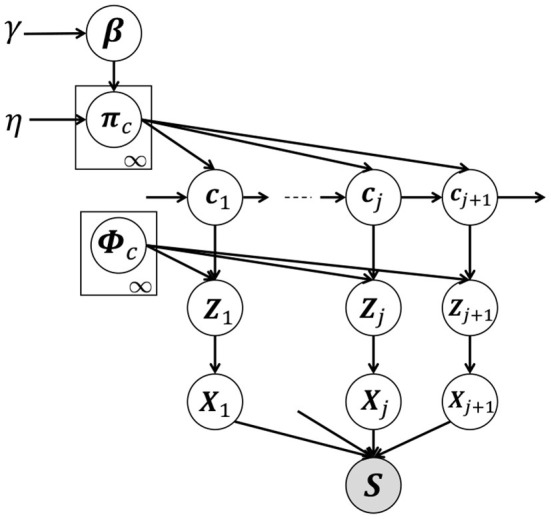
Graphical model of HVGH. The white nodes represent unobserved variables, and the gray node represents the high-dimensional observed sequence that is obtained by concatenating segments.

Figure 3 shows a graphical model of our proposed HVGH, which is a generative model of time-series data. In this model, *c*_*j*_(*j* = 1, 2, ··· , ∞) denotes the classes of the segments, where the number of classes is assumed to be countably infinite. β denotes an infinite-dimensional multinomial distribution, which is generated from the GEM distribution (Pitman, 2002), parameterized by γ. GEM denotes the co-authors Griffiths, Engen, and McCloskey—with the so-called stick-breaking process (SBP) (Sethuraman, 1994). The probability ***π***_*c*_ denotes the transition probability, which is generated by the Dirichlet process (Teh et al., 2006), parameterized by β:

(1) $$\begin{array}{l} \beta \sim \text{GEM}\left(\gamma \right), \end{array}$$

(2) $$\begin{array}{l} \pi_{c}\sim \text{DP}\left(\eta ,\beta \right). \end{array}$$

γ and η are the concentration parameters of the Dirichlet processes; the smaller the value of the concentration parameter, the sparser the generated distribution is. The process by which the probability distribution is constructed through a two-phase Dirichlet process is called a hierarchical Dirichlet process (HDP) (Teh et al., 2006). HDP is described in detail in Nagano et al. (2018).

The class *c*_*j*_ of the *j*-th segment is determined by the class of the (*j* − 1)-th segment and transition probability ***π***_*c*_. The segment of latent variables ***Z***_*j*_ is generated by a Gaussian process (MacKay, 1998) with the parameter ϕ_*c*_ as follows:

(3) $$\begin{array}{l} c_{j}\sim P\left(c|c_{j-1},\pi_{c}\right), \end{array}$$

(4) $$\begin{array}{l} Z_{j}\sim GP\left(Z|\phi_{c_{j}}\right), \end{array}$$

where ϕ_*c*_ represents the parameter of the Gaussian process corresponding to the class *c* and is a set of segments classified into the class *c* in the learning phase. The segment ***X***_*j*_ is generated from the segment of the latent variables ***Z***_*j*_ by using the decoder *p*_*dec*_ of the VAE:

(5) $$\begin{array}{l} X_{j}\sim p_{dec}\left(X|Z_{j}\right). \end{array}$$

The observed sequence ***s*** = ***X***_1_, ***X***_2_, ··· , ***X***_*J*_ is assumed to be generated by connecting segments ***X***_*j*_ sampled by the above generative process. Similarly, the sequence of the latent variables $\bar{s}=Z_{1},Z_{2},\cdot \cdot \cdot \;,Z_{J}$ is obtained by connecting the segments of the latent variables ***Z***_*j*_. In this paper, the *i*-th data point included in ***X***_*j*_ is described as ***x***_*ji*_, and the *i*-th data point included in ***Z***_*j*_ is described as ***z***_*ji*_. If the characters represent what they obviously are, we omit their subscripts.

The generative process of the observed sequence ***s*** described above is summarized in Algorithm 1. This pseudo code represents the generative process, and it is difficult to directly implement this code because the number of classes is infinite. The details on how to address this problem are described in section 3.

**Algorithm 1:** Generative process of *s* by HVGH

1: Draw β ~ GEM(γ)
2: **for** *c* = 1, ··· , ∞ **do**
3: Draw ***π***_*c*_ ~ DP(η, β)
4: **end for**
5:
6: ***s*** = ϵ (an empty sequence)
7: **for** *j* = 1, ··· , *J* **do**
8: Draw *c*_*j*_ ~ *P*(*c*_*j*_|*c*_*j* − 1_, ***π***_*c*_),
9: Draw $Z_{j}\sim GP\left(Z_{j}|\phi_{c_{j}}\right)$,
10: Draw ***X***_*j*_ ~ *p*_*dec*_(***X***_*j*_|***Z***_*j*_),
11: Append ***X***_*j*_ to ***s***.
12: **end for**

### 2.1. Gaussian Process (GP)

In this paper, each class represents a continuous trajectory by learning the emission *z*_*i*_ of time step *i* using a Gaussian process (MacKay, 1998). In the Gaussian process, given ***t***_*c*_ and ***i***_*c*_, which are the vectors of the latent variable *z*_*i*_ that is classified into class *c* and its time step *i*, respectively (details are explained later), the predictive distribution of ẑ of time step î is a Gaussian distribution with the parameters μ and σ^2^:

(6) $$\begin{array}{l} p\left(\hat{z}|\hat{i},\phi_{c}\right)\propto N\left(z|\mu ,\sigma^{2}\right), \end{array}$$

(7) $$\begin{array}{l} \mu =k^{T}C^{-1}t_{c}, \end{array}$$

(8) $$\begin{array}{l} \sigma^{2}=\rho -k^{T}C^{-1}k. \end{array}$$

Here, ***C*** is a matrix having the following elements:

(9) $$\begin{array}{l} C\left(i_{p},i_{q}\right)=k\left(i_{p},i_{q}\right)+\omega^{-1}\delta_{pq}, \end{array}$$

where *k*(·, ·) denotes the kernel function and ω denotes a hyperparameter that represents noise in the observations. ***k*** is a vector with the elements *k*(*i*_*p*_, î), and ρ is *k*(î, î). ϕ_*c*_ represents a set of the segments of latent variables that are classified into the class *c*, and ***t***_*c*_ and ***i***_*c*_ are the vectors where a respective latent variable *z*_*i*_ and time step *i* in ϕ_*c*_ are arranged. For example, assuming that the latent variables have one dimension, and segments ***Z***_1_, ***Z***_2_, ···  are classified into class *c*, we can compute ***t***_*c*_ and ***i***_*c*_ as follows:

(10) $$\begin{array}{l} \phi_{c}=\left\{Z_{1},Z_{2},\cdot \cdot \cdot \;\right\}=\left\{\left\{z_{11},z_{12},z_{13},\cdot \cdot \cdot \;\right\},\left\{z_{21},z_{22},z_{23},\cdot \cdot \cdot \;\right\},\cdot \cdot \cdot \;\right\}, \end{array}$$

(11) $$\begin{array}{l} t_{c}={\left[z_{11},z_{12},z_{13},\cdot \cdot \cdot \;,z_{21},z_{22},z_{23},\cdot \cdot \cdot \;\right]}^{T}, \end{array}$$

(12) $$\begin{array}{l} i_{c}={\left[1,2,3,\cdot \cdot \cdot 1,2,3,\cdot \cdot \cdot \;\right]}^{T}. \end{array}$$

**Figure 4:**
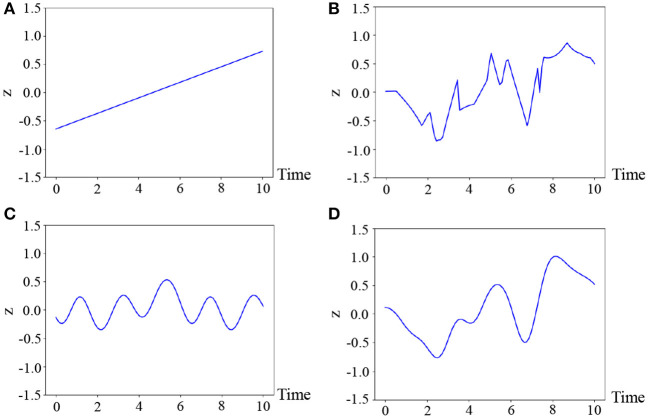
Four temporal patterns sampled from the Gaussian processes, where **(A)** linear : *i*_*p*_*i*_*q*_, **(B)** exponential : exp(−|*i*_*p*_ − *i*_*q*_|), **(C)** periodic : exp(cos|*i*_*p*_ − *i*_*q*_|), and **(D)** RBF : $\exp\left(-|i_{p}-i_{q}{|}^{2}\right)$ are used.

Once ***t***_*c*_ and ***i***_*c*_ are computed, they are shared to compute the probability of (ẑ, î) being classified into class *c*. A Gaussian process can represent complicated time-series data owing to the kernel function. In this paper, we used the following kernel function:

(13) $$\begin{array}{l} k\left(i_{p},i_{q}\right)=\theta_{0}\exp\left(-\frac{1}{2}\theta_{1}{\left\|i_{p}-i_{q}\right\|}^{2}\right)+\theta_{2}+\theta_{3}i_{p}i_{q}, \end{array}$$

where θ_*_ denotes the parameters of the kernel, which are fixed for all classes in our experiments. The reason why we select this kernel is that the motions are generally smooth and, hence, we consider the latent variable to also be temporally smooth. Figure 4 illustrates the samples from various kernels: linear, exponential, periodic, and radial basic function (RBF) kernels (Bishop, 2006). As can be observed in this figure, it is difficult for the linear and periodic kernels to represent a non-linear and non-periodic pattern, and the sample of the exponential kernel is not smooth. On the other hand, the RBF kernel can represent a smooth temporal pattern. Therefore, we use the kernel based on the RBF kernel, which is generally used for Gaussian processes (Bishop, 2006). However, it is not evident as to which kernel is the most appropriate. Moreover, the appropriate kernel depends on the task. This issue will be considered in future work because it exceeds the scope of this paper.

Additionally, if the observations are composed of multidimensional vectors, we assume that each dimension is independently generated. Therefore, the predictive distribution $GP\left(z|\phi_{c}\right)$ by which the emission ***z*** = (*z*^(1)^, *z*^(2)^, ··· ) of time step *i* is generated using a Gaussian process of class *c* is computed as follows:

(14) $$\begin{array}{l} \mathrm{GP}\left(z|\phi_{c}\right)=p\left(z^{\left(1\right)}|i,\phi_{c,1}\right)p\left(z^{\left(2\right)}|i,\phi_{c,2}\right)p\left(z^{\left(3\right)}|i,\phi_{c,3}\right)\cdot \cdot \cdot \end{array}$$

(15) $$\begin{array}{c} \;=N\left(z^{\left(1\right)}|\mu_{1},\sigma_{1}^{2}\right)N\left(z^{\left(2\right)}|\mu_{2},\sigma_{2}^{2}\right)N\left(z^{\left(3\right)}|\mu_{3},\sigma_{3}^{2}\right)\cdot \cdot \cdot \end{array}$$

By using this probability, the latent variables can be classified into the classes. Moreover, because each dimension is independently generated, the mean vector ***μ***_*c*_(*i*) and the variance–covariance matrix Σ_*c*_(*i*) of $\mathrm{GP}\left(z_{ji}|\phi_{c}\right)$ are represented as follows:

(16) $$\begin{array}{l} \mu_{c}\left(i\right)=\left(\mu_{1},\mu_{2},\mu_{3},\cdot \cdot \cdot \;\right), \end{array}$$

(17) $$\begin{array}{l} \Sigma_{c}\left(i\right)=\left[\begin{array}{ccc} \sigma_{1}^{2} & 0 & 0\\ 0 & \sigma_{2}^{2} & 0\\ 0 & 0 & \ddots \end{array}\right], \end{array}$$

where (μ_1_, μ_2_, μ_3_, ··· ) and $\left(\sigma_{1}^{2},\sigma_{2}^{2},\sigma_{3}^{2},\cdot \cdot \cdot \;\right)$, respectively, represent the mean and variance of each dimension. HVGH is a model in which the VAE and GP influence each other mutually with the use of ***μ***_*c*_(*i*) and Σ_*c*_(*i*) as the prior distribution of the VAE.

### 2.2. Overview of the Variational Autoencoder

In this paper, we compress a high-dimensional time-series observation into low-dimensional latent variables using the VAE (Kingma et al., 2013). The VAE is a neural network that can learn the correspondence between a high-dimensional observation ***x*** and the latent variable ***z***. Generally, in a probabilistic model, the posterior distribution of ***z*** can be expressed as follows:

(18) $$\begin{array}{l} p\left(z|x\right)=\frac{p_{dec}\left(x|z\right)p\left(z\right)}{p\left(x\right)}. \end{array}$$

**Figure 5:**
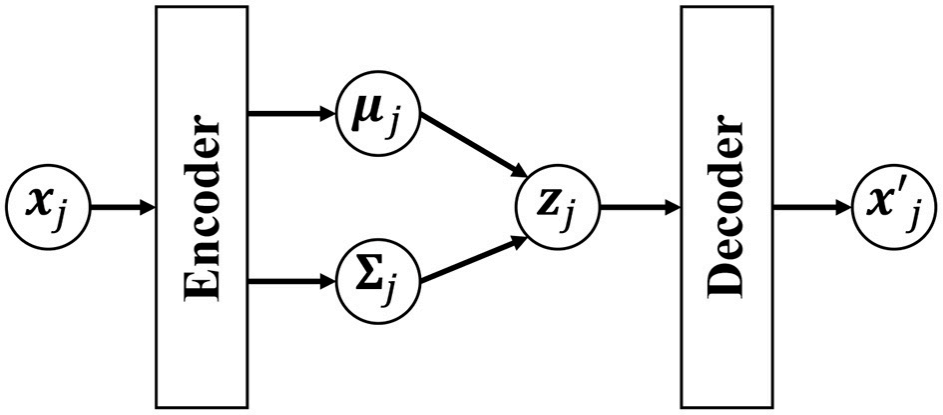
Overview of the variational autoencoder (VAE). The low-dimensional latent variable ***z***_*j*_ is obtained by compressing the observed data point ***x***_*j*_ through the encoder network. $x_{j}^{\prime }$ is an observation reconstructed by the decoder from the latent variable ***z***_*j*_.

However, if a neural network that has expressive power is used for the generative model *p*_*dec*_(***x***|***z***), Equation (18) cannot be analytically derived. To solve this problem, in the VAE, *p*(***z***|***x***) is approximated by *q*_*enc*_(***z***). Figure 5 shows an overview of the VAE. A Gaussian distribution with a mean ***μ***_*enc*_(***x***) and variance Σ_*enc*_(***x***) that are estimated by using encoder networks from input ***x*** is used as *q*_*enc*_(***z***):

(19) $$\begin{array}{l} q_{enc}\left(z\right)=N\left(z|\mu_{enc}\left(x\right),\Sigma_{enc}\left(x\right)\right). \end{array}$$

The latent variable ***z*** is stochastically determined by this distribution, and ***x***′ is generated through decoder networks *p*_*dec*_:

(20) $$\begin{array}{l} z\sim q_{enc}\left(z\right), \end{array}$$

(21) $$\begin{array}{l} x^{\prime }\sim p_{dec}\left(x|z\right). \end{array}$$

The parameters of the encoder and decoder are determined to maximize the likelihood by using the variational Bayesian method. Generally, the prior distribution of the VAE is a Gaussian distribution, the mean of which is the zero vector 0, and the variance–covariance matrix is the identity matrix ***e***. On the other hand, HVGH uses a Gaussian distribution whose parameters are ***μ***_*c*_(*i*) and Σ_*c*_(*i*) of class *c* into which ***z***_*ji*_ is classified. As a result, latent space suitable for segmentation can be constructed. By using this VAE, a sequence of the observation ***s*** = ***X***_1_, ***X***_2_, ··· , ***X***_*J*_ is converted into a sequence of the latent variables $\bar{s}=Z_{1},Z_{2},\cdot \cdot \cdot \;,Z_{J}$ through the encoder.

## 3. Parameter Inference

The log likelihood of HVGH is expressed as follows:

(22) $$\begin{array}{l} \log p\left(X_{1},\cdot \cdot \cdot \;,X_{J},c_{1},\cdot \cdot \cdot \;,c_{J}\right) \end{array}$$

(23) $$\begin{array}{l} \;=\log\underset{j}{\prod }\int_{Z_{j}}p\left(Z_{j},c_{j}\right)p\left(X_{j}|Z_{j}\right)dZ_{j} \end{array}$$

(24) $$\begin{array}{l} \;=\log\underset{j}{\prod }\int_{Z_{j}}\underset{HDP-GP-HSMM}{\underset{︸}{\mathrm{GP}\left(Z_{j}|\phi_{c}\right)P\left(c_{j}|c_{j-1},\pi_{c}\right)}}\underset{VAE}{\underset{︸}{p\left(X_{j}|Z_{j}\right)}}dZ_{j}. \end{array}$$

**Figure 6:**
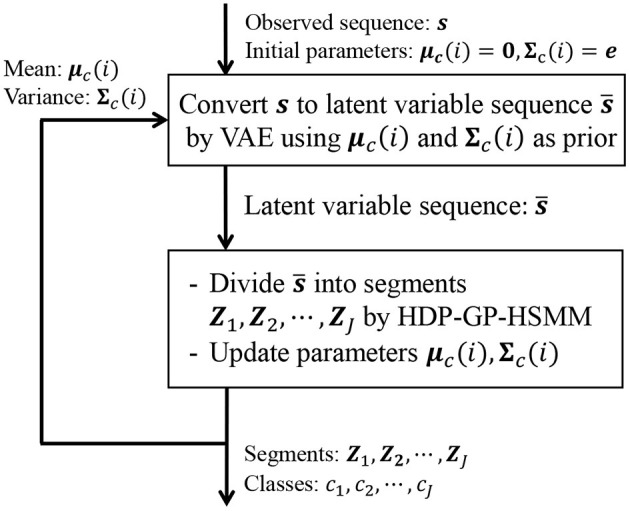
Overview of parameter estimation for HVGH. The parameters are learned by a mutual learning loop of the VAE and HDP-GP-HSMM.

In Equation (24), the first and second factors are computed in HDP-GP-HSMM, and the third factor is computed in VAE. It is difficult to directly maximize Equation (22); therefore, HDP-GP-HSMM and the VAE are alternately optimized, and the parameters that approximately maximize Equation (22) are computed. Figure 6 depicts an outline of the parameter estimation for HVGH. A sequence of observations ***s*** = ***X***_1_, ***X***_2_, ··· , ***X***_*J*_ is converted into a sequence of latent variables $\bar{s}=Z_{1},Z_{2},\cdot \cdot \cdot \;,Z_{J}$ by the VAE. Then, through HDP-GP-HSMM, the sequence of latent variables $\bar{s}$ is divided into segments of latent variables ***Z***_1_, ***Z***_2_, ··· , ***Z***_*J*_, and the parameters ***μ***_*c*_(*i*) and Σ_*c*_(*i*) of the predictive distribution of ***z*** are computed. This predictive distribution is used as a prior distribution of the VAE. Thus, the parameters of the VAE and HDP-GP-HSMM are mutually optimized.

### 3.1. Parameter Inference of HDP-GP-HSMM

#### 3.1.1. Blocked Gibbs Sampler

In HDP-GP-HSMM, segments and classes of latent variables are determined by sampling. For efficient estimation, we utilize a blocked Gibbs sampler (Jensen et al., 1995), in which all segments and their classes in one observed sequence are sampled. First, all sequences of the latent variables are randomly divided into segments and randomly classified into classes. Next, the segments of latent variables ***Z***_*nj*_(*j* = 1, 2, ··· , *J*_*n*_) obtained by segmenting the *n*-th sequence ${\bar{s}}_{n}$ are excluded from the training data, and the parameters of the Gaussian process ϕ_*c*_ and transition probabilities *P*(*c*|*c*′) are updated. The segments of latent variables and their classes are sampled as follows:

(25) $$\begin{array}{l} \left(Z_{n,1},\cdot \cdot \cdot \;,Z_{n,J_{n}}\right),\left(c_{n,1},\cdot \cdot \cdot \;,c_{n,J_{n}}\right)\sim p(\left(Z_{1},Z_{2},\cdot \cdot \cdot \;,Z_{J}\right),\\ \;\left(c_{1},c_{2},\cdot \cdot \cdot \;,c_{J}\right)|{\bar{s}}_{n}). \end{array}$$

The parameters of the Gaussian process of each class ϕ_*c*_ and transition probability *P*(*c*|*c*′) are updated by using the sampled segments and their classes. The parameters are optimized by iterating this procedure. Algorithm 2 is the pseudo code of the blocked Gibbs sampler. *N*_*c*_*nj*__ and *N*_*c*_*nj*_, *c*_*n,j*+1__ are parameters to compute the transition probability in Equation (29). However, it is difficult to compute Equation (25) because an infinite number of classes is assumed. To overcome this problem, we use a slice sampler to compute these probabilities by stochastically truncating the number of classes.

Moreover, the probabilities of all possible patterns of segments and classifications are required in Equation (25), and these cannot be computed naively owing to the large computational cost. To compute Equation (25) efficiently, we utilize forward filtering–backward sampling (Uchiumi et al., 2015).

**Algorithm 2:** Blocked Gibbs Sampler

1: Repeat until convergence
2: **for** *n* = 1 to *N* **do**
3: **for** *j* = 1 to *J*_*n*_ **do**
4: *N*_*c*_*nj*__− = 1
5: *N*_*c*_*nj*_, *c*_*n,j*+1__− = 1
6: Delete segments ***Z***_*nj*_ from ϕ_*c*_*nj*__
7: **end for**
8: // Sampling segments and their classes
9: ***Z***_n*_, *c*_n*_ ~ *p*(Z_*_, c_*_|***s***_*n*_)
10: **for** *j* = 1 to *J*_*n*_ **do**
11: *N*_*c*_*nj*__ + +
12: *N*_*c*_*nj*_, *c*_*n,j*+1__ + +
13: Append segments ***Z***_*nj*_ to ϕ_*c*_*nj*__
14: **end for**
15: **end for**

#### 3.1.2. Slice Sampler

**Figure 7:**
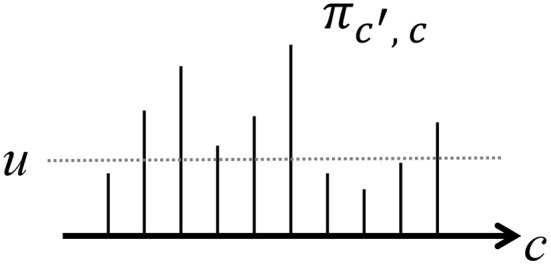
Slice sampling truncates the number of classes by thresholding $\pi_{c^{\prime },c}$.

In the HDP, we assumed that the number of classes is countably infinite. However, it is difficult to compute Equation (25) because *c* can have infinite possibilities. To overcome this problem, we use a slice sampler (Van Gael et al., 2008) to stochastically truncate the number of classes. In the slice sampler, an auxiliary variable *u*_*j*_ that follows the distribution for each time step *j* is introduced:

(26) $$\begin{array}{l} p\left(u_{j}|c_{j-1},c_{j}\right)=\frac{\xi \left(0<u_{j}<\pi_{c_{j-1},c_{j}}\right)}{\pi_{c_{j-1},c_{j}}}, \end{array}$$

where ξ(*A*) = 1 if condition *A* is true; otherwise, it is 0. By truncating the classes with a transition probability π_*c*_*j*−1_, *c*_*j*__ that is less than *u*_*j*_, the number of classes becomes finite, as shown in Figure 7.

#### 3.1.3. Forward Filtering–Backward Sampling

The number of classes can be truncated by slice sampling. Consequently, forward filtering–backward sampling (Uchiumi et al., 2015) can be applied to compute Equation (25). In forward filtering, the probability that *k* samples ${\bar{s}}_{t-k:k}$ before time step *t* form a segment of class *c* is as follows:

(27) $$\begin{array}{l} \alpha \left[t\right]\left[k\right]\left[c\right]\\ =GP\left({\bar{s}}_{t-k:k}|\phi_{c}\right)P_{len}\left(k|\lambda \right)\overset{K}{\underset{k^{\prime }=1}{\sum }}\overset{\bar{C}}{\underset{c^{\prime }=1}{\sum }}\left\{P\left(c|c^{\prime },\beta ,\eta \right)\alpha \left[t-k\right]\left[k^{\prime }\right]\left[c^{\prime }\right]\right\}, \end{array}$$

where $\bar{C}$ denotes the maximum number of classes estimated by slice sampling and *K* denotes the maximum length of segments. *P*_*len*_(*k*|λ) represents a Poisson distribution with a mean parameter λ:

(28) $$\begin{array}{l} P_{len}\left(k|\lambda \right)=\frac{\lambda^{k}e^{-\lambda }}{k!}. \end{array}$$

**Figure 8:**
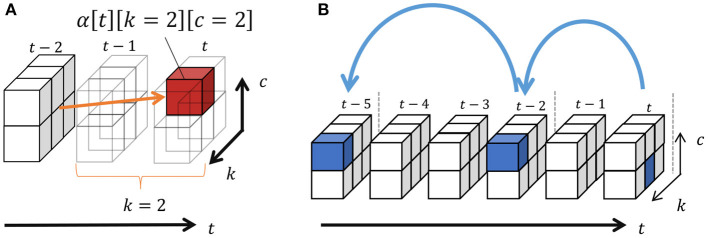
**(A,B)** Forward filtering–backward sampling.

This corresponds to the distribution of the segment lengths. In addition, *P*(*c*|*c*′, β, η) is the transition probability, which can be computed as follows:

(29) $$\begin{array}{l} P\left(c|c^{\prime },\beta ,\eta \right)=\frac{N_{c^{\prime }c}+\eta \beta_{c^{\prime }}}{N_{c^{\prime }}+\eta }, \end{array}$$

where $N_{c^{\prime }}$ represents the number of segments of class *c* and $N_{c^{\prime }c}$ denotes the number of transitions from *c*′ to *c*. In addition, *k*′ and *c*′ are the length and class of possible segments before ${\bar{s}}_{t-k:k}$, respectively, and these probabilities are marginalized out in Equation (27). Moreover, α[*t*][*k*][*] = 0 if *t* − *k* < 0, and α[0][0][*] = 1.0. Equation (27) can be recursively computed from α[1][1][*] using dynamic programming, as shown in Figure 8A. This figure depicts an example of computing α[*t*][2][2], which is the probability that the two samples before *t* become a segment having the class *c*. In this case, the length is two and, therefore, all the segments with end points *t* − 2 can potentially transit to this segment. In α[*t*][2][2], these possibilities are marginalized out. Finally, the length of segments and their classes can be sampled through backward sampling from *t* = *T*:

(30) $$\begin{array}{l} k,c\sim p\left(z_{j}|s_{t-k:t}\right)\alpha \left[t\right]\left[k\right]\left[c\right]P\left(c_{j-1}|c\right). \end{array}$$

Figure 8B depicts an example of backward sampling. The length of segment *k*_1_ and its class *c*_1_ are sampled from the probabilities α[*t*][*][*]. If *k*_1_ = 2, *k*_2_ and *c*_2_ are sampled from α[*t* − 2][*][*]. By iterating this procedure until *t* = 0, the segments and their classes can be determined. Algorithm 3 shows the pseudo-code of forward filtering–backward sampling with slice sampling.

**Algorithm 3:** Forward Filtering–Backward Sampling

1: // Slice sampling
2: **for** *j* = 1 to *J*_*n*_ **do**
3: *u*_*j*_ ~ *p*(*u*_*j*_|*c*_*j*−1_, *c*_*j*_)
4: **end for**
5: $\bar{C}=\underset{j}{\max}\left(\text{count}\left(\pi_{c_{j-1},c_{j}}>u_{j}\right)\right)$
6: // Forward filtering
7: **for** *t* = 1 to *T* **do**
8: **for** *k* = 1 to *K* **do**
9: **for** *c* = 1 to $\bar{C}$ **do**
10: Compute α[*t*][*k*][*c*]
11: **end for**
12: **end for**
13: **end for**
14: // Backward sampling
15: *t* = *T, j* = 0, *c*_0_ = 0
16: **while** *t* > 0 **do**
17: *j* = *j* + 1
18: *k, c* ~ *p*(***z***_*j*_|***s***_*t*−*k*:*t*_)α[*t*][*k*][*c*]*P*(*c*_*j*−1_|*c*)
19: ***z***_*j*_ = ***s***_*t*−*k*:*t*_
20: *c*_*j*_ = *c*
21: *t* = *t* − *k*
22: **end while**
23: *J*_*n*_ = *j*
24: return (***z***_*J*_*n*__, ***z***_*J*_*n*_−1_, ··· , ***z***_1_), (*c*_*J*_*n*__, *c*_*J*_*n*_−1_, ··· , *c*_1_)

### 3.2. Parameter Inference of the VAE

The parameters of the encoder and decoder of VAE are estimated to maximize the likelihood *p*(***x***). However, it is difficult to maximize the likelihood directly. Instead, the normal VAE maximizes the following variational lower limit:

(31) $$\begin{array}{l} L\left(x_{ji},z_{ji}\right)=\int q_{enc}\left(z_{ji}|x_{ji}\right)\log p_{dec}\left(x_{ji}|z_{ji}\right)dz_{ji}\\ \;-D_{KL}\left(q_{enc}\left(z_{ji}|x_{ji}\right)\|p\left(z_{ji}|\underline{0},e\right)\right), \end{array}$$

where ∫ *q*_*enc*_(***z***_*ji*_|***x***_*ji*_) log *p*_*dec*_(***x***_*ji*_|***z***_*ji*_)*d**z***_*ji*_ represents the reconstruction error. Moreover, $p\left(z_{ji}|\underline{0},e\right)$ is a prior distribution of ***z***_*ji*_, which is a Gaussian distribution whose mean is 0, and the variance–covariance matrix is ***e***. $D_{KL}\left(q_{enc}\left(z_{ji}|x_{ji}\right)||p\left(z_{ji}|\underline{0},e\right)\right)$ is the Kullback–Leibler divergence, and this functions as a regularization term. On the other hand, in HVGH, the mean ***μ***_*c*_(*i*) and the variance–covariance matrix Σ_*c*_(*i*) are used as the parameters of the prior distribution. These are the parameters of the predictive distribution of class *c* into which ***z***_*ji*_ is classified, and they are estimated by HDP-GP-HSMM:

(32) $$\begin{array}{l} L\left(x_{ji},z_{ji}\right)=\int q_{enc}\left(z_{ji}|x_{ji}\right)\log p_{dec}\left(x_{ji}|z_{ji}\right)dz_{ji}\\ \;-D_{KL}\left(q_{enc}\left(z_{ji}|x_{ji}\right)\|p\left(z_{ji}|\mu_{c}\left(i\right),\Sigma_{c}\left(i\right)\right)\right). \end{array}$$

**Figure 9:**
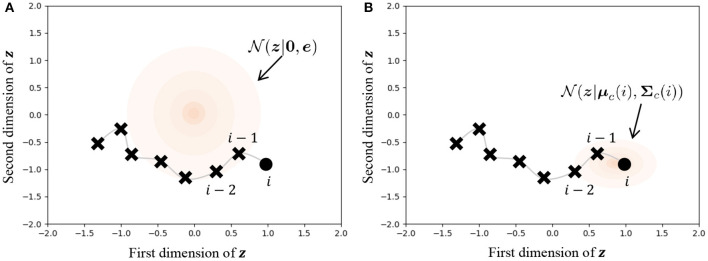
Influence of prior distribution. The orange region represents the standard deviation. **(A)** The same prior distribution is used for any data points in the normal VAE. **(B)** The distribution is varied depending on the time step and the class of the data point in the VAE in HVGH.

Figure 9 illustrates the difference in prior distributions between Equations (31, 32). In the normal VAE using Equation (31), the prior distribution is $N\left(\underline{0},e\right)$ against all data points, as shown in Figure 9A. On the other hand, the parameters of the prior distribution of HVGH are computed by the Gaussian process, as shown in Figure 9B. Because the GP restricts data points that have closer time steps to being more similar values, *z*_*ji*_ becomes a similar value to *z*_*j,i*−1_ and *z*_*j,i*+1_. Therefore, the latent space learned by the VAE can reflect the characteristics of time-series data. Moreover, these parameters have different values depending on the class of the data point. Therefore, the latent space can also reflect the characteristics of each class.

## 4. Experiments

To validate the proposed HVGH, we applied it to several types of time-series data. For comparison, we used HDP-GP-HSMM (Nagano et al., 2018), HDP-HMM (Beal et al., 2002), HDP-HMM+NPYLM (Taniguchi et al., 2011), BP-HMM (Fox et al., 2011), and Autoplait (Matsubara et al., 2014) as baseline methods.

### 4.1. Experimental Setup

To evaluate the validity of the proposed method, we used the following four motion-capture datasets.

**Figure 10:**
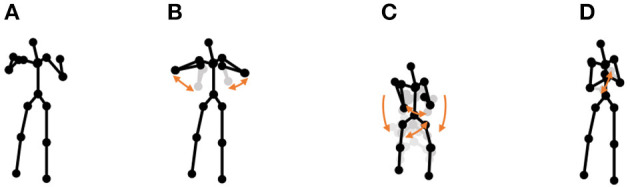
Four unit motions included in the chicken dance: **(A)** beaks, **(B)** wings, **(C)** tail feathers, and **(D)** claps.

**Figure 11:**
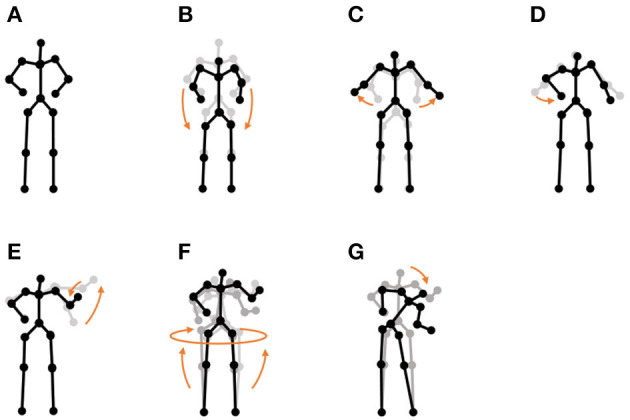
Seven unit motions included in the “I'm a little teapot” dance: **(A)** short and stout, **(B)** bending knee, **(C)** spread arms, **(D)** handle, **(E)** spout, **(F)** steam up, and **(G)** pour.

**Figure 12:**
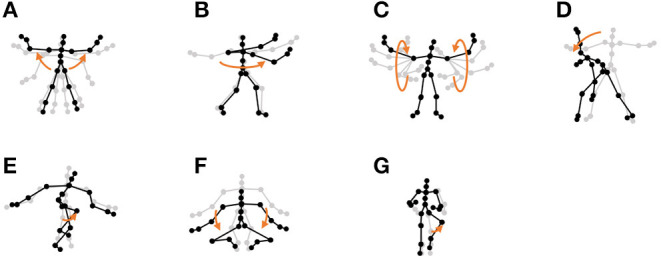
Seven unit motions included in exercise motion 1: **(A)** jumping jack, **(B)** twist, **(C)** arm circle, **(D)** bend over, **(E)** knee raise, **(F)** squatting, and **(G)** jogging.

**Figure 13:**
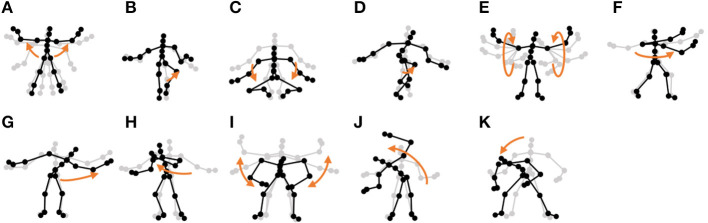
Eleven unit motions included in exercise motion 2: **(A)** jumping jack, **(B)** jogging, **(C)** squatting, **(D)** knee raise, **(E)** arm circle, **(F)** twist, **(G)** side reach, **(H)** boxing, **(I)** arm wave, **(J)** side bend, and **(K)** toe touch.

- **Chicken dance:** We used a sequence of motion-capture data of a human performing a chicken dance from the CMU Graphics Lab Motion Capture Database^2^. The dance includes four motions, as shown in Figure 10.
- **“I'm a little teapot” dance (teapot dance):** We also used two sequences from the teapot dance motion-capture data from subject 29 in the CMU Graphics Lab Motion Capture Database^3^. These sequences include seven motions, as shown in Figure 11.
- **Exercise motion 1:** To determine the validity against more complicated motions, we used three sequences of exercise motion-capture data from subject 13 in the CMU Graphics Lab Motion Capture Database. These sequences include seven motions, as shown in Figure 12.
- **Exercise motion 2:** Furthermore, we used three sequences of different exercises from the motion-capture data from subject 14 in the CMU Graphics Lab Motion Capture Database. These sequences include 11 motions, as shown in Figure 13.

To reduce computational cost, all the sequences were preprocessed by down sampling to 4 fps. These motion-capture datasets included the directions of 31 body parts, each of which was represented by a three-dimensional Euler angle. Therefore, each frame was constructed in 93-dimensional vectors. We used sequences of 93-dimensional vectors as input. Moreover, HVGH requires hyperparameters, and we set them to λ = 14.0, θ_0_ = 1.0, θ_1_ = 1.0, θ_2_ = 0.0, and θ_3_ = 16.0, which were empirically determined for the segmentation of the 4-fps sequences. For the chicken dance exclusively, we set λ to half that of the others because its motion-capture data was shorter than the others. To train the VAE, we used 1/4 of all the data points as a mini batch, Adam (Kingma et al., 2017) was used for the optimization, and the optimization was iterated 150 times. To train HDP-GP-HSMM, the blocked Gibbs sampler was iterated 10 times to converge the parameters. Furthermore, the mutual learning loop of the VAE and HDP-GP-HSMM was iterated until the variational lower limit converged.

### 4.2. Evaluation Metrics

To evaluate the segmentation accuracy, we used four measures: the normalized Hamming distance, precision, recall, and F-measure.

The normalized Hamming distance represents the error rate of the classification of the data points, and it is computed as follows:

(33) $$\begin{array}{l} ND\left(c,\bar{c}\right)=\frac{D\left(c,\bar{c}\right)}{\left|\bar{c}\right|}, \end{array}$$

where ***c*** and $\bar{c}$, respectively, represent sequences of estimated classes and correct classes in the data points in the observed sequence. Moreover, $D\left(c,\bar{c}\right)$ represents the Hamming distance between two sequences, and $\left|\bar{c}\right|$ is the length of the sequence. Therefore, the normalized Hamming distance ranges from zero to one, and smaller values indicate that the estimated classes are more similar to the ground truth.

**Figure 14:**
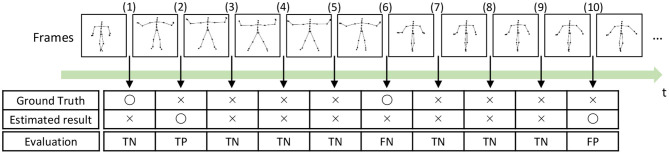
Example of segmentation evaluation: TP is assigned to the boundary (2) because the estimated boundary is within the error range from the true boundary.

To compute the precision, recall, and F-measure, we evaluated boundary points (boundaries between segments) as true positives (TPs), true negatives (TNs), false positives (FPs), and false negatives (FNs), as shown in Figure 14. A TP is assigned to the points that are correctly estimated as boundary points. An estimated boundary point is treated as correct if the estimated boundary is within the error range, as shown in Figure 14, Frame (2). The error range is defined as ±ψ% of the sequence length, and ψ represents the percentage of the error range. A TN is assigned to the points that are correctly estimated not to be boundary points, as shown in Figure 14, Frame (3). Conversely, FPs and FNs are assigned to points that are falsely estimated as boundary points, as shown in Figure 14, Frame (10), and falsely estimated not to be boundary points, as shown in Figure 14, Frame (6), respectively. From these boundary evaluations, the precision, recall, and F-measure are computed as follows:

(34) $$\begin{array}{l} P=\frac{N_{TP}}{N_{TP}+N_{FP}}, \end{array}$$

(35) $$\begin{array}{l} R=\frac{N_{TP}}{N_{TP}+N_{FN}}, \end{array}$$

(36) $$\begin{array}{l} F=\frac{2P\cdot R}{P+R}, \end{array}$$

where *N*_*TP*_, *N*_*FP*_, and *N*_*FN*_ represent the number of boundary points estimated as TP, FP, and, FN, respectively.

**Figure 15:**
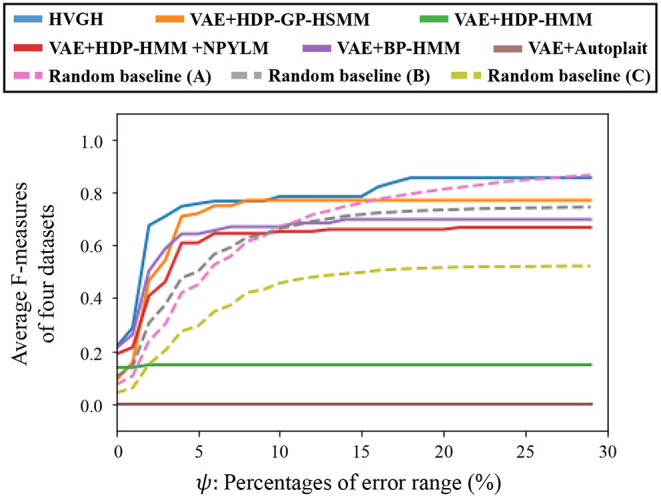
Variation of F-measure with the error range.

**Figure 16:**
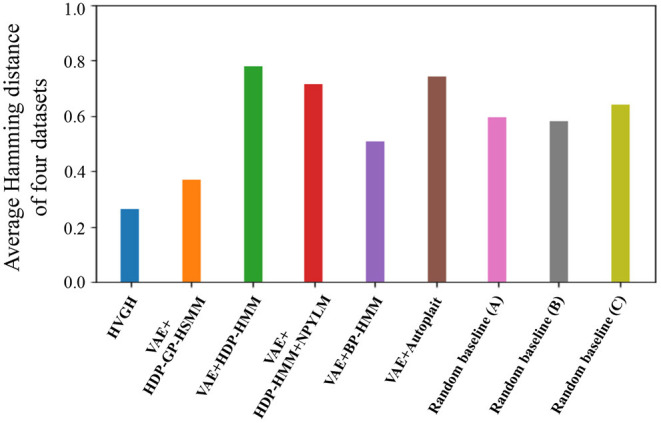
Hamming distance of each method.

Figure 15 depicts the results of a preliminary experiment to determine the error range. The horizontal axis represents the percentages of error range ψ, and the vertical axis represents the average F-measures of the segmentation of four datasets used in the experiments. The details of the datasets are described in section 4.1. Figure 16 shows the result of the average Hamming distance of the four datasets used in the experiments.

To support the evaluation, we used three random baseline methods (A-C). The random baselines were computed given the number of segments as follows: a sequence is divided into the specified number of segments by using a uniform distribution, and classes of the segments are randomly sampled from the uniform distribution. The random baselines (A-C) represent the results of baseline segmentation using the correct number of segments, double the number, and half the number, respectively. The sequences are divided by iterating this procedure 100 times, and the values in the figures represent the averages of the 100 segmentation trials. As shown in Figure 15, in a smaller error range, the F-measure of the random baseline (B) is greater than that of the random baseline (A). This is because the number of boundary points of the random baseline (B) is greater than that of the random baseline (A), the more boundary points of (B) are likely to be within the error range, and TP increases. On the other hand, in a larger error range, the F-measure of the random baseline (B) is less than that of the random baseline (A). This is because the more boundary points of the random baseline (A) are also within the error range in the case of a larger error range, and TP increases. Moreover, the precision of the random baseline (B) decreases and recall increases with an increase in FP because the number of boundary points is greater than that for the random baseline (A). In contrast, the F-measure of the random baseline (C) is less than that of the random baseline (A). This is because precision increases and recall decreases with an increase in FN. In the case of the percentages of error range where the F-measure is saturated, the F-measure is lower in both cases in which the number of segments is larger or smaller because of the trade-off relationship between recall and precision. These results indicate that F-measure reflects the accuracy of boundary points as well as the correctness of the number of segments. From Figure 15, we can see that the F-measure begins to saturate at the 5% error range in all methods except for the random baselines; therefore, we use a 5% error range in the subsequent experiments.

### 4.3. Segmentation of Motion-capture Data

First, we applied baseline methods to the 93-dimensional time-series data. However, the baseline methods were not able to segment the 93-dimensional time-series data appropriately, because of high dimensionality. Therefore, we applied the VAE with the same parameters as HVGH, and sequences of three-dimensional latent variables were used for segmentation of the baseline methods. Tables 1–4 show the results of segmentation on each of the four motion-capture datasets. The random baselines in the tables indicate the results of the random segmentation, which are described in section 4.2.

**Table 1:** Segmentation results for the chicken dance.

	**Hamming distance**	**Precision**	**Recall**	**F-measure**	**# of estimated classes**
HVGH	0.23	0.86	0.86	0.86	4
VAE + HDP-GP-HSMM	0.31	1.0	0.71	0.83	4
VAE + HDP-HMM	0.73	0.15	1.0	0.26	11
VAE + HDP-HMM+NPYLM	0.74	0.85	0.79	0.81	15
VAE + BP-HMM	0.33	1.0	0.86	0.92	3
VAE + Autoplait	0.66	0.0	0.0	0.0	1
Random baseline (A)	0.55	0.55	0.41	0.47	4
Random baseline (B)	0.51	0.45	0.69	0.54	4
Random baseline (C)	0.60	0.64	0.20	0.30	3

**Table 2:** Segmentation results for the teapot dance.

	**Hamming distance**	**Precision**	**Recall**	**F-measure**	**# of estimated classes**
HVGH	0.31	0.74	0.83	0.79	7
VAE + HDP-GP-HSMM	0.36	0.80	0.71	0.75	8
VAE + HDP-HMM	0.75	0.10	1.0	0.17	16
VAE + HDP-HMM+NPYLM	0.61	0.58	1.0	0.74	21
VAE + BP-HMM	0.34	0.50	0.86	0.63	10
VAE + Autoplait	0.75	0.0	0.0	0.0	1
Random baseline (A)	0.59	0.54	0.46	0.49	6
Random baseline (B)	0.59	0.42	0.72	0.53	7
Random baseline (C)	0.65	0.59	0.21	0.31	5

**Table 3:** Segmentation results for the exercise motion 1: subject 13.

	**Hamming distance**	**Precision**	**Recall**	**F-measure**	**# of estimated classes**
HVGH	0.27	0.66	0.93	0.75	14
VAE + HDP-GP-HSMM	0.41	0.53	0.93	0.67	11
VAE + HDP-HMM	0.79	0.05	1.0	0.09	10
VAE + HDP-HMM+NPYLM	0.76	0.32	1.0	0.48	34
VAE + BP-HMM	0.57	0.29	1.0	0.45	7
VAE + Autoplait	0.76	0.0	0.0	0.0	2
Random baseline (A)	0.60	0.45	0.38	0.41	7
Random baseline (B)	0.59	0.36	0.62	0.46	7
Random baseline (C)	0.64	0.51	0.21	0.29	5

**Table 4:** Segmentation results for the exercise motion 2: subject 14.

	**Hamming distance**	**Precision**	**Recall**	**F-measure**	**# of estimated classes**
HVGH	0.23	0.50	1.0	0.66	13
VAE + HDP-GP-HSMM	0.39	0.46	1.0	0.63	14
VAE + HDP-HMM	0.82	0.03	1.0	0.07	25
VAE + HDP-HMM+NPYLM	0.75	0.23	0.86	0.36	42
VAE + BP-HMM	0.79	0.48	0.81	0.55	4
VAE + Autoplait	0.79	0.0	0.0	0.0	3
Random baseline (A)	0.62	0.46	0.41	0.43	9
Random baseline (B)	0.63	0.37	0.66	0.47	11
Random baseline (C)	0.66	0.51	0.19	0.28	7

VAE+HDP-GP-HSMM and VAE+BP-HMM were able to segment the motion-capture data from the chicken dance and teapot dance. However, in the results with exercise motion obtained using VAE+BP-HMM, the value of the normalized Hamming distance was larger and the F-measure was smaller than those for the dance motions. This is because simple and discriminative motions were repeated in the chicken dance and teapot dance. Therefore, BP-HMM, which is an HMM-based model, was able to segment them. In contrast, the Gaussian process used in HVGH and HDP-GP-HSMM is non-parametric, making it possible to represent complicated motion patterns in the exercise data. Moreover, HVGH achieved more accurate segmentation than HDP-GP-HSMM. We believe that this is because the appropriate latent space for the segmentation was constructed by using the predictive distribution of the GP as the prior distribution of the VAE in HVGH.

Furthermore, the number of motion classes in the chicken dance and teapot dance was correctly estimated by HVGH. In the exercise motion, larger numbers were estimated because their sequences included complicated motions. In the case of exercise motion 1, 14 classes were estimated by HVGH—more than the correct number seven. This is because the stationary state was estimated as a unit of motion, and symmetrical motion was separately classified as left-sided and right-sided motion in different classes. Moreover, 13 classes—more than the correct number 11—were estimated by HVGH in exercise motion 2. Again, this is because stationary motion was estimated as one motion and because the symmetrical motion shown in Figure 13J was divided into two classes: left- and right-sided motion. However, it is reasonable to estimate the stationary state as a unit of motion. Further, dividing a symmetrical motion into two classes was not erroneous, because the observed values for the left- and right-sided motion were different. Therefore, we conclude that HVGH yielded better results in this case.

**Figure 17:**
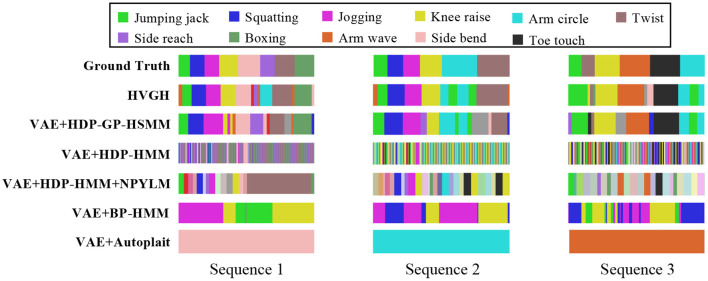
Segmentation results for exercise motion 2.

Figure 17 illustrates the segmentation results for exercise motion 2. In this graph, the horizontal axis represents time steps, the color reflects motion classes, and the top bar is the ground truth of the segmentation. It is clear that the segments and their classes estimated by HVGH are the most similar to the ground truth.

Moreover, we compared the VAE with other dimensional compression methods in HDP-GP-HSMM. Table 5 presents the results of the segmentation of exercise motion 2 obtained using the methods in which dimensional compression is performed through principal component analysis (PCA) (Pearson, 1901) and independent component analysis (ICA) (Hyvärinen et al., 2000) instead of VAE. PCA and ICA are generally used for dimensional compression. We used the general FastICA^4^ as the ICA implementation, and their three-dimensional output was used identical to the latent variables of VAE. In the case of PCA and ICA, the min-max normalization, in which the values are normalized to a range from –1 to 1, was applied for the sequence of latent variables, as with Nagano et al. (2018).

**Table 5:** Segmentation results of comparison with other compression methods.

	**Hamming distance**	**Precision**	**Recall**	**F-measure**	**# of estimated classes**
HVGH	0.23	0.50	1.0	0.66	13
VAE + HDP-GP-HSMM	0.39	0.46	1.0	0.63	14
PCA + HDP-GP-HSMM	0.49	0.44	0.77	0.56	7
ICA + HDP-GP-HSMM	0.56	0.48	0.66	0.54	7

Additionally, we described the results of HVGH and VAE+HDP-GP-HSMM for comparison. PCA and ICA compress the high-dimensional data into low-dimensional data by linear transform, and complicated high-dimensional time-series data cannot be converted into low-dimensional data appropriate for segmentation by using these methods. On the other hand, VAE non-linearly compresses high-dimensional data by using a neural network and enables the conversion of the high-dimensional time-series data into low-dimensional data appropriate for segmentation.

**Figure 18:**
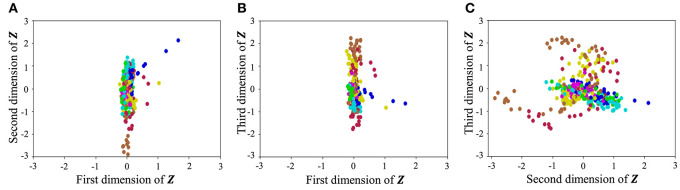
Latent space of the VAE: **(A–C)** respectively represent the first and second, first and third, and second and third dimension of the latent variables. The color of each point, which is latent variable reflects the correct motion class.

**Figure 19:**
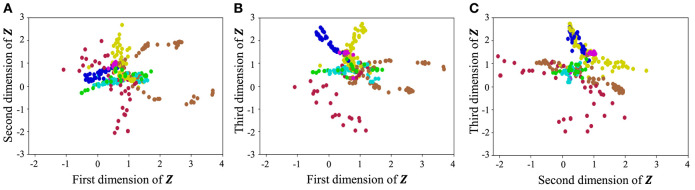
Latent space of the HVGH: **(A–C)** respectively represent the first and second, first and third, and second and third dimension of the latent variables. The color of each point, which is latent variable reflects the correct motion class.

With regard to exercise motion 1, Figure 18 shows the latent variables estimated by the VAE, and Figure 19 shows the latent variables learned by mutual learning with HVGH. In these figures (a-c), respectively, represent the first and second, first and third, and second and third dimensions of the latent variables. The color of each point reflects the correct motion class. In Figure 18, latent variables do not necessarily reflect the motion class, because they were estimated with the VAE exclusively. In contrast, in Figure 19, the latent variables in the same class have more similar values. This means that latent space is estimated to represent the features of unit motions. However, it is difficult to fully understand the meaning of latent space because the VAE represents a non-linear relationship between high-dimensional output and low-dimensional latent variables. Thus, we qualitatively analyze them by comparing the latent variables with motions, where each dimension roughly represents the following motions:

- **Positive region in the first dimension:** right and left motions of the arms
- **Negative region in the first dimension:** up and down motions of the arms
- **Positive region in the second dimension:** twisting of the upper body to the left
- **Negative region in the second dimension:** twisting of the upper body to the right
- **Positive region in the third dimension:** motions of the legs
- **Negative region in the third dimension:** bending of the upper body

From these results, we conclude that HVGH can estimate the correct number of classes and accurate segments from high-dimensional data by using the proposed mutual learning loop.

## 5. Conclusion

In this paper, we proposed HVGH, which segments high-dimensional time-series data by mutual learning of a VAE and HDP-GP-HSMM. In the proposed method, high-dimensional vectors are converted into low-dimensional latent variables representing features of unit motions with the VAE. Using these latent variables, HVGH achieves accurate segmentation. The experimental results showed that the segments, their classes, and the number of classes could be estimated correctly using the proposed method. Moreover, the results showed that HVGH is effective with various types of high-dimensional time-series data compared to a model where the VAE and HDP-GP-HSMM are used independently.

However, the computational cost of HVGH is very high because it takes *O*(*N*^3^) to learn *N* data points using a Gaussian process, and this is repeated in the mutual learning loop. Because of this problem, HVGH cannot be applied to large-scale time-series data. We plan to reduce the computational cost by introducing the approximation method for the Gaussian process proposed in Nguyen-Tuong et al. (2009) and Okadome et al. (2014).

Moreover, to simplify the computation, we assumed that the dimensions of the observation were independent, and we consider this assumption reasonable because the experimental results demonstrated that the proposed method works well. However, the dimensions of the observation are not actually independent, and the dependency between the dimensions will need to be considered to model more complicated whole-body motion. We believe that multi-output Gaussian processes can be used to represent dependencies between dimensions (Álvarez et al., 2010; Dürichen et al., 2014).

In HVGH, we do not consider the time warp of the time-series data. Although GP can deal with motions whose speeds are slightly different by estimating the variance, motions whose speeds are considerably different are classified into different classes. Therefore, we will investigate the robustness of HVGH against time warp in a future study and extend it to a method considering time warp.

## Data Availability Statement

Publicly available datasets were analyzed in this study. This data can be found here: http://mocap.cs.cmu.edu/.

## Author Contributions

MN, TNak, TNag, and DM conceived, designed, and developed the research. MN and TNak performed the experiment and analyzed the data. MN wrote the manuscript with support from TNak, TNag, DM, and IK. DM, IK, and WT supervised the project. All authors discussed the results and contributed to the final manuscript.

### Conflict of Interest

The authors declare that the research was conducted in the absence of any commercial or financial relationships that could be construed as a potential conflict of interest.

## Footnotes

^1^HVGH stands for hierarchical Dirichlet process, variational autoencoder, Gaussian process, and hidden semi-Markov model.

^2^http://mocap.cs.cmu.edu/: subject 18, trial 15.

^3^http://mocap.cs.cmu.edu/: subject 29, trials 3 and 8.

^4^https://scikit-learn.org/stable/modules/generated/sklearn.decomposition.FastICA.html
